# Er:YAG Laser for Brackets Bonding: A SEM Study after Debonding

**DOI:** 10.1155/2014/935946

**Published:** 2014-10-22

**Authors:** G. Ierardo, G. Di Carlo, F. Petrillo, V. Luzzi, I. Vozza, G. Migliau, R. Kornblit, J. P. Rocca, A. Polimeni

**Affiliations:** ^1^Department of Oral and Maxillofacial Science, “Sapienza” University of Rome, Via Caserta 6, 00161 Rome, Italy; ^2^Faculty of Odontology, University Hospital “St. Roch”, University of Nice-Sophia Antipolis, 5 rue Pierre Dévoluy, 06006 Nice, France

## Abstract

*Background*. The introduction of Er:YAG laser in dentistry for ablation of hard tissues advocated an alternative method of enamel etching for orthodontics purpose. *Materials and Methods*. 55 extracted human third molars were inserted in acrylic resin blocks and divided into five groups of 11 teeth. Group 1 was treated with 37% orthophosphoric acid for 30 seconds. Group 2 was treated with laser irradiation (Er:YAG Fidelius III, Fotona, Slovenia) at 80 mJ and 4 Hz. Group 3 underwent laser treatment (80 mJ, 4 Hz), followed by 37% orthophosphoric acid for 30 seconds. The teeth in Group 4 were treated with laser at 40 mJ and 10 Hz. The teeth in Group 5 were treated with laser (40 mJ, 10 Hz), followed by 37% orthophosphoric acid for 30 seconds. The adhesive remnant index was determined after debonding. *Results*. Kruskas-Wallis test showed that location parameters (median and mean) are significantly different between Groups 2 and 4 when compared with control group; on the contrary no significant difference was detected between Groups 3 and 5 with the controls. *Conclusion*. The use of Er:YAG laser alone, as in Groups 2 and 4, showed no significant advantages over phosphoric acid in the bonding procedure for orthodontics brackets.

## 1. Introduction

Phosphoric acid etching is the gold standard method of enamel preparation before application of bonding resins for orthodontic brackets [1]. Enamel etching changes the tooth surface from being of low-energy and hydrophobic to being of high-energy and hydrophilic, increasing the surface area for bonding [2]. Studies have demonstrated that this kind of attachment can have disadvantages, such as enamel decalcification, which leaves the enamel surface susceptible to acid attack (cavity formation) under orthodontic brackets [3–6]. One of the most important challenges in orthodontic treatment, however, is the frequent debonding of brackets, with the consequent lengthening of treatment duration.

With the recent introduction of erbium-doped yttrium aluminum garnet (Er:YAG) laser in dentistry for the ablation of hard tissues, including enamel and dentin, laser enamel preparation has been proposed as an alternative to phosphoric acid etching [7–9]. The Er:YAG laser can effectively modify enamel and dentin surfaces because of its 2.94 mm wavelength emission, which coincides with the main absorption band of water and OH− groups in hydroxyapatite [10].

In dentistry, the Er:YAG laser is primarily used to ablate hard tissues (enamel, dentin, and bone), but also to treat soft tissues [11–14]. Many papers [15–17] have reported that Er:YAG laser ablation of enamel and dentin is effective and efficient without causing heat damage to the pulp and without carbonization or cracks in the irradiated enamel and dentin. Moreover, use of the Er:YAG laser for dental hard tissue treatment, such as caries removal, cavity preparation, and enamel etching within certain parameters, is both safe and effective [18–21]. Additionally, the surface created by laser etching is reportedly resistant to carious attacks [22]. One study reported that the ultrastructural morphological changes in the surface enamel of permanent teeth after irradiation with Er:YAG laser were similar to lava flow, with an opened prism core and modification of the prism form [23]. To evaluate the advantages of the Er:YAG laser for enamel surface preparation before orthodontic bracket bonding, this study compared the adhesive remnant index (ARI) scores of teeth treated with different bonding procedures.

## 2. Materials and Methods

Our study included 55 intact human third mandibular and maxillary molars, extracted for orthodontic reasons. The inclusion criteria were noncarious lesions or enamel defects. The teeth were stored in saline solution at 4°C for no more than 28 days before insertion into acrylic resin blocks. The teeth were then divided in five groups of 11 teeth each. The first group (control group) was treated with 37% orthophosphoric acid (etching solution, ORMCO, USA) for 30 seconds. The second group was treated with laser irradiation (Er:YAG Fidelius III, Fotona, Slovenia) at 80 mJ and 4 Hz. The third group underwent laser treatment (80 mJ and 4 Hz), followed by 37% orthophosphoric acid for 30 seconds. The teeth in the fourth group were treated with laser at 40 mJ and 10 Hz. The fifth group underwent laser treatment (40 mJ and 10 Hz), followed by 37% orthophosphoric acid for 30 seconds.

**Figure 1:**
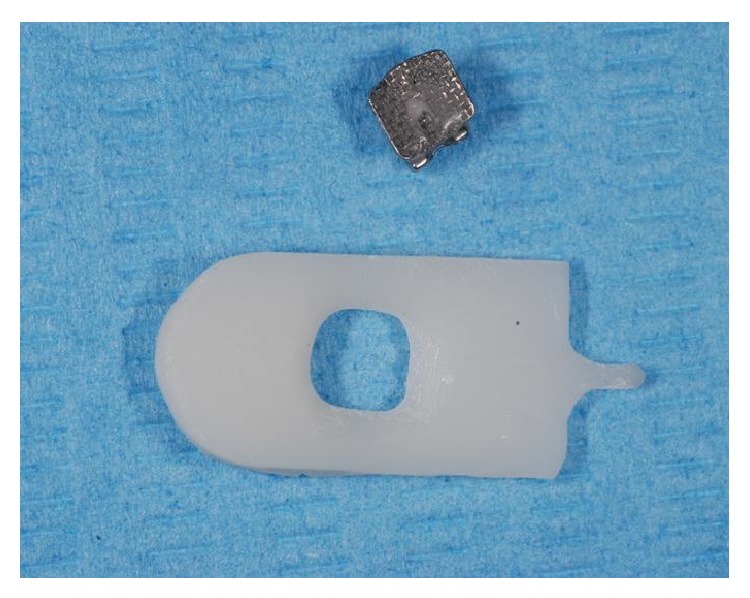
Ceramic mask equipped with the central hole of the size corresponding to the bracket base.

**Figure 2:**
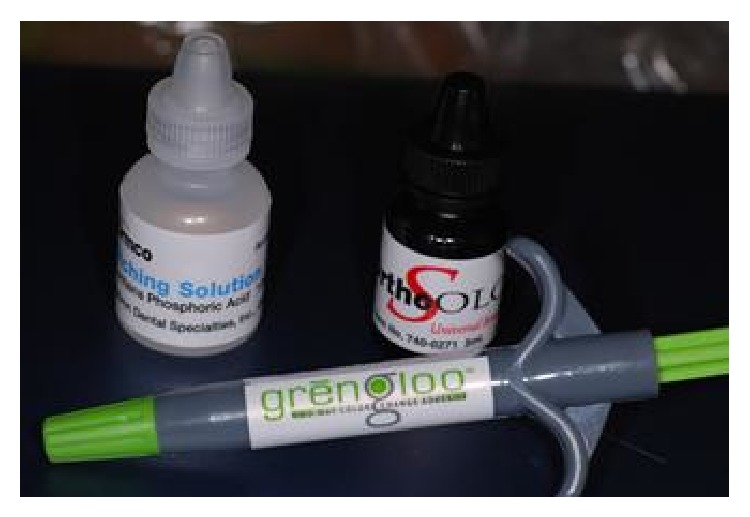
Phosphoric acid, bonding and composite resin.

**Figure 3:**
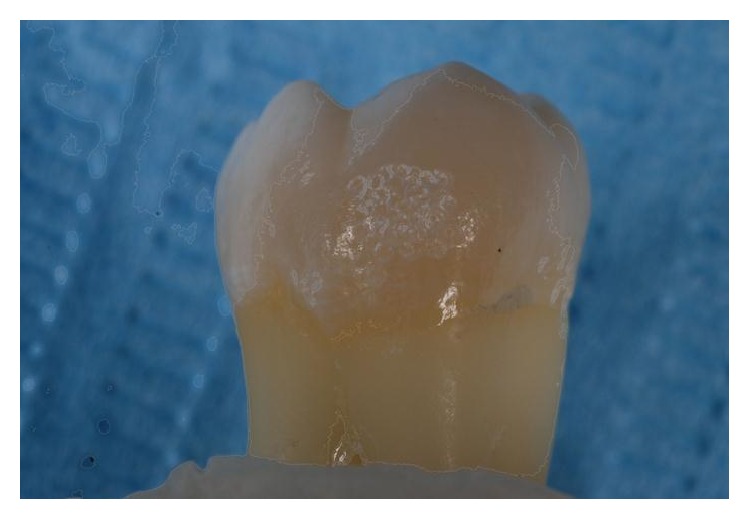
Enamel surface after conditioning with Er:YAG laser.

**Figure 4:**
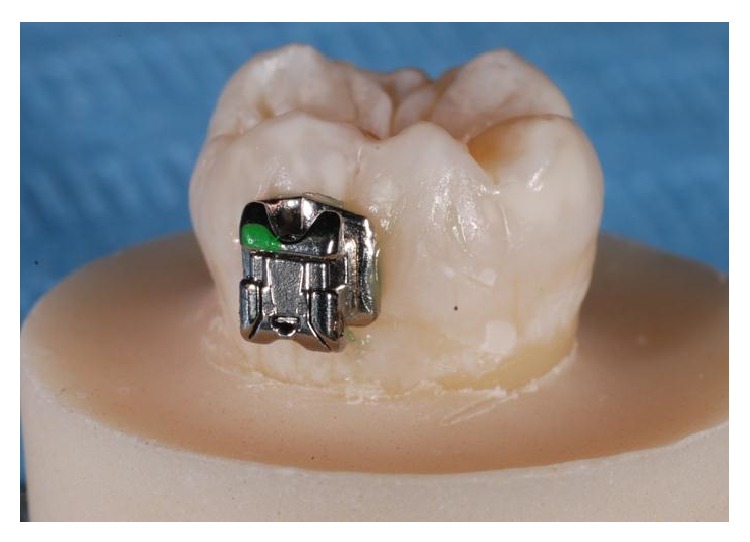
Bracket bonded on the enamel.

**Figure 5:**
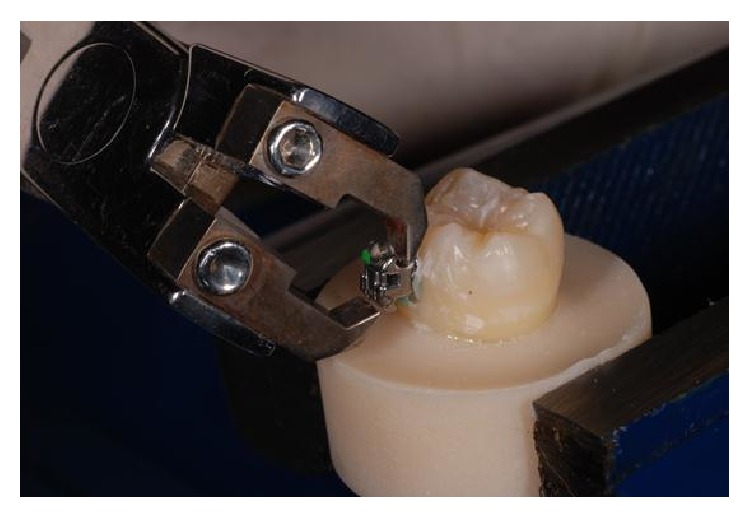
The sample stabilized by a vice, during the debonding.

To limit the area of enamel treated, a ceramic window was prepared with the exact dimensions of an orthodontic bracket. The ceramic window was held on the tooth surface by one operator while a second one applied the acid or laser light treatment only to the area within the window (Figure 1). The Er:YAG laser was used with the following parameters: VSP mode (pulse length, 100 *μ*s) with the noncontact handpiece (mirror) in a focus mode (theoretical distance from the tooth surface, 10 mm) using water/air spray in a continuous movement on a theoretical spot 0.8 mm in diameter (one spot next to another). The same operator (R. Kornblit) performed all* laser* enamel conditioning under 2.5 × 350 magnification (Univet medical eyewear). Immediately after enamel surface preparation, a bracket (Damon MX3-UR3, ORMCO) was attached by an experienced orthodontist (G. Ierardo) to each tooth following the different procedure for each single group as explained above. All teeth were dried before bonding placement. The bonding was performed using the same bonding adhesive (ORTHOSOLO, ORMCO) and a composite material (GRENGLOO, ORMCO) (Figures 2, 3, and 4). A microbrush was used to apply adhesive for 10 seconds on each surface, followed immediately by a thin layer of composite resin and a bracket. Teeth were cured for 30 seconds with a Coolbeam Orthodontic Curing Light (ORMCO). The bonded teeth were then kept in saline solution in five different plastic boxes at room temperature for 48 hours to allow complete polymerization. After 48 hours, all brackets were manually removed from the 55 teeth by the same experienced orthodontist (G. Ierardo), using a debonding plier (AEZ 8664008, ORMCO) designed for this procedure and exerting continuous rotational force toward the cervical part of the tooth (Figure 5). All 55 teeth were then sectioned in vertical (mesiodistal) and horizontal (cervical) directions with an abrasive disc (COD: Yellow-Flex 220) by the same operator who performed the laser preparation (R. Kornblit).

All 55 samples were dipped for 30 seconds in an ultrasonic bath at 30°C to remove any residual powder left after sectioning. The samples were then kept in an oven at 40°C for 24 hours to remove all moisture, which can interfere with the vacuum needed for metallization. All samples were then conventionally metallized (Gold sputtering JEOL JFC 1100E) and observed under scanning electron microscope (SEM) (JEOL, JSM 5310 LV).

The ARI score was recorded by a senior student who was not informed regarding the different procedure applied for each tooth using a stereoscope (Nikon, Tokyo, Japan) at 10x magnification to determine the amount of residual adhesive remaining on each tooth, as described by Contreras-Bulnes et al. [24]. ARI scores were recorded using the 5-point scale described by Bishara and Trulove [25, 26]: 1 = no composite adhering to the bracket base, 2 = adhered composite on less than 10% of the bracket, 3 = adhered composite on more than 10% but less than 90% of the bracket, 4 = adhered composite on more than 90% of the bracket, and 5 = composite adhering to the entire bracket base.

### 2.1. Statistical Analysis

The statistical analysis aims at testing whether location parameters for variable “ARI score” are statistically different between each treatment and the control group.

ARI score is an ordered categorical variable, so nonparametric statistics are used. Thus, median, interquartile difference, and Kruskal-Wallis rank sum test [27, 28] are used in place of mean, standard error, and ANOVA, which are suitable for numeric and normally distributed variables.

At first a graphical analysis is performed, drawing boxplots of “ARI score” in control group and in each treatment.

Secondly a single group analysis is performed, computing descriptive statistics (median, 1st and 3rd quartile) in each group (Table 1).

Thirdly the null hypothesis that the medians are the same in each group is tested against the alternative that they differ in at least one group by the Kruskal-Wallis rank sum test. As the null hypothesis is rejected, the after Kruskal-Wallis multiple comparison test between treatments versus control is performed.

## 3. Results

The macroscopic observation of the composition material on the tooth surface was as follows.

- Group 1: 6 samples presented all the composite that remained on the tooth; in 5 samples part of the composite remained on the tooth and a part on the bracket.
- Group 2: 4 samples presented all the composite that remained on the tooth; in 7 samples part of the composite remained on the tooth and a part on the bracket.
- Group 3: 2 samples presented all the composite that remained on the tooth; in 8 samples part of the composite remained on the tooth and a part on the bracket and 1 sample presented all the composite that remained on the brackets.
- Group 4: 4 samples presented all the composite that remained on the tooth; in 2 samples part of the composite remained on the tooth and a part on the bracket and 5 samples presented all the composite that remained on the brackets.
- Group 5: 5 samples presented all the composite that remained on the tooth; in 5 samples part of the composite remained on the tooth and a part on the bracket and 1 sample presented all the composite that remained on the brackets.

**Figure 6:**
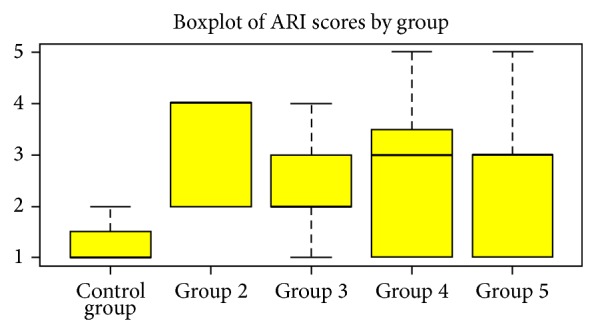


The descriptive statistics regarding ARI score for each single group is presented in Table 1. No cracks were observed under SEM in any of the 55 samples. Boxplots highlight that all treatments show higher location and dispersion towards control group (Figure 6), so significant differences are expected. The null that location parameters of “ARI scores” are the same in each group is rejected, as the Kruskal-Wallis rank sum test is 13.8863 and its *P* value is 0.007667. The Kruskal-Wallis multiple comparison test show that the null is rejected at 5% significance level when comparing Group 2 and Group 4 to control and at 1% significance level when comparing Group 2 and control (Table 2).

## 4. Discussion

ARI score results showed that the best composite resin retention to the enamel surface occurred in the control group (Group 1), in which the enamel surfaces were prepared with acid etching alone. Groups 3 and 5, in which the enamel surfaces were treated with laser before acid etching, had better retention of the composite material to the tooth surface compared with Groups 2 and 4. This finding can be explained by the fact that laser irradiation destroys enamel prisms in an indifferent way: the core of the prism as well as the walls is destroyed (Class 3 in the Silverstone classification), resulting in the typical lava flow appearance of the enamel surface under SEM [20, 21]. This surface is poorly wettable by the bonding material; when phosphoric acid is applied to this surface, the acid attacks and regularizes the enamel's surface, increasing the microinfiltration capacity of the bonding material.

No cracks were observed at the periphery of the bracket attachment in any of the 55 samples, confirming that debonding forces did not damage the enamel surface. This lack of damage probably resulted from the use of an appropriate adhesive system and a specific instrument designed for bracket debonding. Moreover, for samples in which part of the composite remained on the tooth surface and part on the bracket, the group treated with laser alone did not have as homogeneous an adhesive area after debonding as that of the samples treated with acid. This finding confirmed the Silverstone Class 3 classification of enamel surfaces treated with Er:YAG laser.

Bishara and Trulove believed that bond failure at the enamel-adhesive interface was preferable to failure at other locations, because it leaves less residual adhesive and consequently requires less chair time for removal [25]. Several years later, the same author demonstrated that bond failure at the bracket-adhesive surface was better than at the enamel-adhesive interface, because it reduced the risk of enamel fracture and crazing during debonding [26]. We found that acid etching produced the most instances of debonding at the bracket-adhesive surface. We believe that reducing the risk of damage to the pulp is crucial when the debonding procedure is applied in orthodontics.

Authors have reported that laser etching is a valuable method comparable to the classical acid etching procedure [29–32]. These studies were based on shear bond strength measurements; the different laser irradiation protocol makes comparisons with the present study difficult. Moreover, as stated by Contreras-Bulnes et al., this method can result in substantial enamel loss [24]. Our results support those of Martínez-Insua et al., who reported that adhesion to dental hard tissues after Er:YAG laser etching is inferior to that obtained after conventional acid etching [33].

## 5. Conclusion

The use of Er:YAG laser* alone* showed no significant advantages over phosphoric acid etching in the bonding procedure for orthodontic brackets. Taking into account the cost and the additional time required to use the laser, this technology does not currently represent an added value for orthodontists in improving resin adhesion.

## Figures and Tables

**Table 1:** ARI score in whole sample and within groups—descriptive statistics.

Group	*n*. obs.	Min.	1st Q.	Median	3rd Q.	Max.
Control group	11	1	1	1	1,5	2
Group 2	11	2	2	4	4	4
Group 3	11	1	2	2	3	4
Group 4	11	1	1	3	3,5	5
Group 5	11	1	1	3	3	5
Whole sample	55	1	1	2	3	5

**Table 2:** Results of Kruskal-Wallis multiple comparison test, treatment groups versus control (two-tailed).

Comparisons	Observed diff.	*P* value					
		10%		5%		1%	
		Critical diff.	Difference	Critical diff.	Difference	Critical diff.	Difference
Control group—Group 2	23,636	15,312	TRUE	17,063	TRUE	20,653	TRUE
Control group—Group 3	14,909	15,312	FALLS	17,063	FALLS	20,653	FALLS
Control group—Group 4	17,273	15,312	TRUE	17,063	TRUE	20,653	FALLS
Control group—Group 5	12,364	15,312	FALLS	17,063	FALLS	20,653	FALLS
